# Monitoring recurrent angioedema: Findings from the Turkish angioedema control test validation study

**DOI:** 10.1002/clt2.12342

**Published:** 2024-02-28

**Authors:** Semra Demir, Deniz Eyice‐Karabacak, Emek Kocatürk, Derya Ünal, İlkim Deniz Toprak, Pelin Korkmaz, Ayşe Feyza Aslan, Işıl Göğem İmren, Bahar Dikicier, Nevzat Kahveci, Nida Öztop, Rabia Öztaş Kara, Halim İşsever, Marcus Maurer, Karsten Weller, Aslı Gelincik

**Affiliations:** ^1^ Division of Immunology and Allergy Diseases Department of Internal Medicine Istanbul Faculty of Medicine Istanbul University Istanbul Turkey; ^2^ Department of Dermatology Koç University Faculty of Medicine Istanbul Turkey; ^3^ Institute of Allergology Charite‐Universitatsmedizin Corporate Member of Freie Universitat Berlin and Humboldt‐Universitat zu Berlin Berlin Germany; ^4^ Fraunhofer Institute for Translational Medicine and Pharmacology ITMP, Immunology and Allergology Berlin Germany; ^5^ Faculty of Medicine Department of Dermatology Sakarya University Sakarya Turkey; ^6^ Adult Allergy and Immunology Clinic Başakşehir Pine and Sakura City Hospital Istanbul Turkey; ^7^ Istanbul Faculty of Medicine Department of Public Health Istanbul University Istanbul Turkey

**Keywords:** angioedema, angioedema control test, control, disease activity, hereditary angioedema, impact, minimal clinically important difference, quality of life, reliability, tools, validity

## Abstract

**Background:**

Determination of control level in recurrent angioedema (RAE) is necessary to guide management. Here, we validated a Turkish version of the angioedema control test (AECT) for 4‐week (AECT‐4wk) and for 3‐month (AECT‐3mth) and assessed their utility in monitoring RAE.

**Method:**

The recommended structured translation process for patient‐reported outcome measures was completed. The final versions were administered to 51 patients with mast cell‐mediated angioedema (MMAE) and 38 patients with hereditary angioedema, and the minimal clinically important difference (MCID) was determined. Additionally, anchor surveys comprising angioedema activity score for 28 days (AAS‐28 day), visual analog score for angioedema control, Likert scale for the control level from the patient's perspective (LS‐AEC), angioedema quality of life, short form‐12 (SF‐12) and patients' assessment of treatment sufficiency were applied.

**Results:**

The Turkish AECT versions showed good convergent validity with a substantial correlation with anchor tools and known‐group validity. Excellent internal consistency and reproducibility were observed. Equal or more than 10 of 16 points scored with the AECT‐4wk and AECT‐3mth identified patients with well‐controlled disease. The disease activity, control and burden parameters were consistent with the disease control level defined depending on the cut‐off point 10 of AECT. Three‐point changes in AECT‐4wk and ‐3 mt could detect MCID in disease control in all patients.

**Conclusions:**

Turkish AECT versions are valid and reliable tools for assessing and monitoring disease control in patients with RAE. The use of the Turkish versions of the AECT in routine patient care, clinical trials and angioedema research is recommended.

## 1 INTRODUCTION

Recurrent angioedema (RAE) is characterized by repeated intermittent, localized, self‐limiting swelling of the subcutaneous and/or submucosal tissue.^1^ RAE can be bradykinin‐mediated (BMAE), mast cell‐mediated (MMAE), or due to unknown mechanisms.^2^, ^3^

Patients with recurrent MMAE, which was previously defined as histaminergic angioedema, often also have itchy wheals (hives), but recurrent MMAE can also be isolated.^2^ Recurrent MMAE with or without wheals, by definition, is a clinical manifestation of chronic urticaria.^3^

BMAE can be acquired or hereditary and is characterized by increased levels of bradykinin, which induces increased vascular permeability and vasodilatation by activating the bradykinin 2 receptor.^4^ As for acquired subtypes of recurrent BMAE, angiotensin converting enzyme inhibitors and dipeptidyl peptidase IV inhibitors can lead to impaired degradation of bradykinin, and autoimmune and lymphoproliferative diseases can result in decreased levels of C1 inhibitor (C1 INH) and in increased production of bradykinin. Most cases of bradykinin‐driven hereditary angioedema (HAE) are due to defects in the SERPING1 gene leading to low serum levels and decreased function of C1 inhibitor in HAE‐C1INH type 1 and type 2, respectively. Bradykinin‐mediated HAE, in patients with normal antigenic or functional levels of C1 INH, can be due to pathogenic variants in factor 12 gene (HAE‐FXII), kininogen 1 gene (HAE‐KNG1), or plasminogen gene (HAE‐PLG).^5^

RAE, in all types and patients, can come with marked deterioration in quality of life and impaired performance in school and at work, and it often does. RAE also markedly affects the families of patients as health care systems.^6^, ^7^, ^8^ Therefore, effective disease management and treatment are crucial in RAE, making use of the treatment of acute attacks and the prevention of attacks with prophylactic therapies, which differ depending on the underlying RAE pathomechanism.^2^, ^3^

Independent of the RAE type, the aims of treatment are complete disease control, the absence of signs and symptoms, and a normal quality of life. Therefore, determining the disease control levels with a standardized, validated, simple and reliable tool is essential. International guidelines recommend the use of patient‐reported outcome measures (PROMs) to evaluate disease activity, disease control, and impairment of the quality of life in RAE.^2^, ^3^ This includes the angioedema control test (AECT), which was developed and validated as a retrospective instrument to measure the disease control level in patients with RAE.^9^, ^10^

There are two versions of the AECT, where one measures disease control during the last 4 weeks (AECT‐4wk) the other one during the last 3 months (AECT‐3mth). Both AECT versions comprise four questions with five answer options each (scored with 0–4 points). Accordingly, the total AECT score ranged from 0 to 16 points. The higher the score, the higher the level of disease control. A total score <10 points and equal or ≥10 points indicates poorly‐controlled and well‐controlled disease, respectively, with 76% sensitivity and 84% specificity.^10^ Recently, the minimal clinically important difference (MCID) of AECT‐4wk was determined to be three points; however, it has not been assessed for the AECT‐3mth yet.^11^

The AECT is easy to perform and score, valid, and reliable, and it has become the gold standard for assessing disease control and guiding treatment decisions in RAE. It is widely available, in 47 languages including Turkish, but the Turkish AECT versions remain to be assessed for their validity, reliability, screening accuracy, sensitivity to change, and MCID. Therefore, we determined these clinicometric properties of the Turkish AECT‐4wk and AECT‐3mth versions and prospectively assessed them for their clinical utility across different types of RAE, that is, MMAE and BMAE including HAE.

## 2 METHODS

### 2.1 Study design

The study included 89 adult patients with RAE followed up at Istanbul Faculty of Medicine Adult Allergy and Immunology Clinic, Koc University Faculty of Medicine Dermatology Clinic and Sakarya University Faculty of Medicine Dermatology Clinic, all of which are members of Turkish Angioedema Center of Reference and Excellence centers.^12^, ^13^

The study was conducted in two phases: linguistic and content validations. After the completion of the linguistic validation, both tools were administered to patients diagnosed with either MMAE or BMAE.

### 2.2 Patient population

Patients' diagnosis was confirmed before enrollment according to international guidelines.^2^, ^3^ All MMAE patients had acquired RAE, and all BMAE patients had HAE type 1 or type 2. Age and education level were similar between groups. The Female/male ratio was higher in MMAE group than in the HAE group (4.1 vs. 1.53, *p* = 0.039). The detailed demographic and clinical features of the patients are shown in Table S1.

Additionally, anchor surveys comprising angioedema activity score for 28 days (AAS‐28 day), visual analog score for angioedema control (VAS‐AEC), Likert scale for the control level from the patient's perspective (LS‐AEC), angioedema quality of life (AE‐QoL), short form‐12 (SF‐12) and patients' assessment of treatment sufficiency were applied.^1^, ^6^, ^14^, ^15^, ^16^

Meanwhile, all patients were assessed by physician expert on RAE and treated according to current international guidelines throughout the study period.^2^, ^3^

The study was approved by the local ethics committee of Istanbul University and written informed consent was obtained from all participants.

### 2.3 Translation and linguistic validation of the Turkish AECT versions

In order to develop a Turkish version of both AECT‐4wk and AECT‐3mth, the original German versions were translated into Turkish according to the structured translation process with a recommended road map consisting of the forward translation, the production of a “reconciled” version, backward translation, backward translation review, development of a consensus version and cognitive debriefing.

The pre‐final Turkish versions which were developed according to consensus between the original and the Turkish authors were cognitively debriefed in four and three patients with MMAE and BMAE/HAE, respectively. Four of them were females. The median (interquartile range [IQR]) age and elapsed time of interview were 44 (28–59) years and 12 (10–15) minutes, respectively. As a result of debriefing, one word was changed in question three to improve understandability. These final versions (Figure S1) were consented by the original developers and subsequently used for the content validation.

### 2.4 Content validation of the Turkish AECT versions

The patients were assessed for their eligibility in the study at visit zero. If the patients accepted to be a participant, they were informed about the study, the demographic and clinical features were recorded and they were invited for further three visits (visit 1, visit 2 and visit 3). The intervals between visit 0 and 1, visit 1 and 2 (for the assessment of AECT‐4wk) were 4 weeks and visit 1 and visit 3 (for the assessment of AECT‐3mth) was 3 months. The final Turkish versions of AECT‐4wk were applied to patients in visits 1 and 2 (4 weeks interval) and AECT‐3mth was applied to participants in visits 1 and 3 (3 months interval).

### 2.5 Assessment of disease activity, quality of life and disease control with other tools

AAS‐28 day which was validated into Turkish, is a kind of angioedema diary that enables the patients self‐record of angioedema attacks once daily.^6^ In visit 0 and visit 1, patients were asked to complete the angioedema activity score for 28 days (AAS‐28 day) for the next visits to evaluate the disease activity. In addition to AAS‐28 day, patients were asked for self‐assessment of their disease control level for the last 4 weeks and 3 months with visual analog scale‐angioedema control (VAS‐AEC‐4wk, VAS‐AEC‐3mth) and Likert scale‐angioedema control for the last 4 weeks and 3 months (LS‐AEC‐4wk, LS‐AEC‐3mth). In VAS‐AEC, patients were asked to mark a point on a continuous line ranging from 0 to 10 points. Zero points indicated that the disease was totally under control, while 10 points indicated poor disease control with maximum complaints.^15^ LS‐AEC is a 5‐point scale and indicates the global level of disease control comprising “completely controlled”, “well‐controlled”, “moderately controlled” “hardly controlled” and “not at all controlled”(10). The treatment sufficiency from the perspective of patients was evaluated with a two‐answer scale including “sufficient treatment” and “not sufficient treatment” for both the last 4 weeks and 3 months.^10^

Other anchor tools including a two‐choice scale for the assessment of the sufficiency of treatment from the patients' perspective and short form‐12 (SF‐12) and AE‐QoL surveys for the determination of disease burden were applied to patients in visits 1–3. AE‐QoL is a specific, valid and reliable survey for the assessment of the disease burden on patients with RAE.^6^ It is composed of four domains and 17 questions and the total score ranges from 0 to 100, with a lower score indicating less impairment in QoL. Also, the impairment in the QoL related to RAE is classified as no relevant impairment (≤30.5), mildly impaired (30.6–37.9), moderately impaired (38–50.5) and severely impaired (≥50.6) according to total score.^17^

SF‐12 is a nonspecific survey and is used in chronic diseases to indicate the impairment in the general health status. It is composed of physical component summary (PCS‐12) and mental component summary (MCS‐12) and the total score ranges from 0 to 100 points with higher scores showing better self‐reported health status.^14^

## 3 DATA ANALYSIS

### 3.1 Analysis for validation

#### 3.1.1 Convergent validity

Convergent validity, that is, the extent to which responses on a test show a strong relationship with responses on conceptually similar tests, of the Turkish AECT‐4wk and AECT‐3mth were determined by Spearman's correlation analysis with the AAS‐28 day, VAS‐AEC, LS‐AEC, AE‐QoL and SF‐12 components as anchor tools. Weak, moderate, and strong correlations were defined by correlation coefficients (*r*) of less than 0.3, between 0.3 and 0.5, and more than 0.5, respectively.^10^

### 3.2 Known groups validity

For the determination of known group validity, which shows the ability of the instrument to distinguish the patients with different disease states, we compared AECT versions with both LS‐AEC, impairment level of QoL and the patients' self‐assessment of treatment sufficiency by Kruskal‐Wallis and Mann‐Whitney U tests according to the type of the data.^10^

### 3.3 Internal consistency reliability

For the assessment of internal consistency reliability of the Turkish AECT versions, Cronbach's *α* coefficient was computed and classified as follows: <0.6, 0.6–0.65, 0.65–0.7, 0.7–0.8, 0.8–0.9 and >0.9 were unacceptable, undesirable, minimally acceptable, respectable, excellent and excessive consistency, respectively.^6^

### 3.4 Test‐retest reliability

Evaluation of stability and reproducibility of Turkish AECT versions was conducted in subgroups of patients with stable disease whose identical angioedema control levels according to LS‐AEC‐4wk and ‐3mth was detected in different visits. Interclass correlation coefficient (ICC) was calculated and classified as 0.5–0.7 indicates moderate to good reproducibility and more than 0.7 indicates excellent reproducibility.^18^

### 3.5 Screening accuracy

For the identification of the patients with poorly‐or well‐controlled disease, LS‐AEC‐4wk and ‐3mth were used as anchor instrument. LS‐AEC was further classified as well‐controlled including the original answers of well and completely controlled and poorly‐controlled including the original answers of not at all, hardly and moderately controlled. Receiver operating characteristic (ROC) analysis and the area under the curve (AUC) value were used to show the screening accuracy of the Turkish AECT versions. No discrimination, acceptable, excellent and outstanding screening accuracy was defined when the AUC values were 0.5, 0.7–0.8, 0.8–0.9 and >0.9, respectively.^19^ After that, patients were categorized as poorly‐ and well‐controlled when AECT score ≤9 and ≥10, respectively. The median scores of AAS‐28 day, number of attacks, AE‐QoL and SF‐12 components, impairment level of QoL and treatment sufficiency were compared between these two groups.

### 3.6 Sensitivity to change

To determine the ability of Turkish AECT versions to detect changes in the patients' disease control, we performed Spearman's correlation analysis between the intra‐individual changes of AECT scores and the other tools determined in two visits showing the changes in disease activity (AAS‐28 day) and control level (VAS‐AEC) and impairment in QoL (AE‐QoL, PCS‐12 and MCS‐12) from visit 1–2 for AECT‐4wk and from visit 1–3 for AECT‐3mth. The correlation degree was categorized as defined in the convergent validity analysis. Moreover, a nonparametric *t*‐test was used to show the relationship between the change in AECT scores and change in patient‐self assessment of treatment sufficiency.

### 3.7 Minimal clinically important difference

MCID indicating the meaningful changes in the patient's state over a period of time is an important feature of PROMs. We determined the MCID of Turkish AECT versions by an anchor‐based method using LS‐AEC.^20^ The patients were grouped into individuals with at least one step change in angioedema control level and with no change and also with at least one step of improvement (meaningful improvement) and with no improvement in angioedema control level according to LS‐AEC. To identify the best cut−off point for MCID in AECT, ROC curve analysis was performed.^16^ The AUC value was categorized as told in the screening accuracy part.

### 3.8 Statistical analysis

IBM SPSS statistics version 22 was used for the analysis of the data. Descriptive data were given as number and percentages or median and IQR according to the type of the data. Categorical data were compared with Chi‐square or Fisher's Exact tests depending on the distribution of the data. Continuous variables were compared with Kruskal–Wallis or Mann Whitney *U* test between groups according to the number of groups. Continuous data determined in different visits for the same scale were compared with a nonparametric paired *t* test. Spearman's correlation test was used for the correlation analysis. Statistical significance level was determined when *p* value was greater than 0.05.

## 4 RESULTS

Fifty‐one and 38 patients with MMAE and BMAE (all HAE) were enrolled, respectively. Eight patients did not attend visits 2 and 3. Females comprised 71.9% of the study population. The median (IQR) age and age at onset of symptoms and diagnosis were 34 (23–50.5), 19 (12–37), and 26 (18–42) years, respectively.

### 4.1 Results of validation analysis

#### 4.1.1 Convergent validity

The Turkish AECT‐4wk was strongly correlated with AAS‐28 day, VAS‐AEC‐4wk and AE‐QoL scores and moderately correlated with the number of attacks in the last month and last 3 months, PCS‐12, and MCS‐12. The Turkish AECT‐3mth was strongly correlated with AE‐QoL and VAS‐AEC‐3mth and moderately correlated with AAS‐28 day, number of attacks in the last 3 months, PCS‐12, and MCS‐12 (Table 1). These results demonstrate a good level of convergent validity for both Turkish AECT versions of AECT.

**TABLE 1:** Convergent validity of the Turkish AECT‐4wk and AECT‐3mth.

	AECT‐4wk		AECT‐3mth	
	*p*	*r*	*p*	*r*
AAS‐28 day	<0.001	−0.552	‐	‐
Number of attacks^a^	<0.001	−0.499	<0.001	−0.412
VAS‐AEC‐4wk or‐3mth	<0.001	−0.764	<0.001	−0.532
AE‐QoL	<0.001	−0.677	<0.001	−0.587
MCS‐12	0.002	0.331	<0.001	0.444
PCS‐12	<0.001	0.409	<0.001	0.357

Abbreviations: AAS‐28, angioedema activity score for 28‐day; AECT, angioedema control test; AE‐QoL, angioedema quality of life; MCS‐12, mental component summary; PCS‐12, physical component summary; VAS‐AEC, visual analogue scale‐angioedema control.

^a^ Number of attacks reported for the previous month and in the last 3 months were analyzed with AECT‐4wk and AECT‐3mth, respectively.

#### 4.1.2 Known‐groups validity

The median AECT‐4wk and ‐3mth scores were significantly different in patients with different levels of disease control and impairment of QoL according to the LS‐AEC and AE‐QoL survey (*p* < 0.001). Also, median AECT‐4wk and AECT‐3mth scores were higher in patients who reported that their treatment was sufficient (*p* < 0.001; *p* = 0.012) (Table 2). Taken together, these results demonstrated that both Turkish AECT versions were able to discriminate patients with various disease states and have high known‐group validity.

**TABLE 2:** Known‐group validity of Turkish angioedema control test versions.

Scales	AECT‐4wk				AECT‐3mth			
	Number of patients	Median	IQR	*p*	Number of patients	Median	IQR	*p*
Likert scale‐PSA^a^								
Completely controlled	19	16	14–16	<0.001	11	12	10–14	<0.001
Well controlled	26	10	7–13		18	10	7.75–12	
Moderately controlled	24	7.5	6–10		23	8	6–10	
Poor controlled	17	5	2.5–7.5		28	5	3.25–8	
Uncontrolled	3	4	4‐.		8	4.5	4–6	
Treatment sufficiency‐PSA^a^								
Sufficient	49	12	8–15	<0.001	36	8	6–12	0.012
Insufficient	39	7	5–10		52	6.5	4–9	
Quality of life (AE‐QoL)								
Not impaired	37	13	10–16	<0.001	37	10	7–12.5	<0.001
Mildly impaired	7	9	7–13		7	8	5–10	
Moderately impaired	16	7.5	5–11.5		16	7	4–8	
Severely impaired	28	6	5–8		28	5	3.25–7.75	

Abbreviations: AECT, angioedema control test; AE‐QoL, angioedema quality of life; PSA, patient self‐assessment.

^a^ For the analysis of AECT‐4wk and of AECT‐3mth Likert scale and treatment sufficiency for last 4 week and for last 3 months were used, respectively.

#### 4.1.3 Internal consistency reliability

Both Turkish AECT versions showed excellent internal consistency reliability with a Cronbach's alpha value of 0.89 for the AECT‐4wk and 0.83 for the AECT‐3mth. In separate analyses of the AECT‐4wk and ‐3mth in patients with MMAE or BMAE/HAE, Cronbach's alpha values were 0.87 and 0.81 and 0.9 and 0.86, respectively, indicating excellent disease type‐specific internal consistency.

#### 4.1.4 Test‐retest reliability

The Turkish AECT‐4wk and ‐3mth were analyzed for test‐retest reliability in 37 and 27 patients for whom disease control levels according to LS‐AEC‐4wk and ‐3mth were the same in two different visits with recall periods of 4‐week and 3‐months, respectively. The mean ± SD AECT‐4wk and AECT‐3mth scores were similar in both visits (AECT‐4wk: 10.57 ± 4.65 vs. 10.76 ± 4.66, *p* = 0.577; AECT‐3mth: 8.37 ± 4.1 vs. 9.15 ± 3.68, *p* = 0.076). The ICC values, which were 0.88 and 0.84 in single measures and 0.94 and 0.91 in average measures for AECT‐4wk and ‐3mth, respectively, indicated excellent reproducibility for both Turkish AECT versions.

#### 4.1.5 Screening accuracy

**FIGURE 1:**
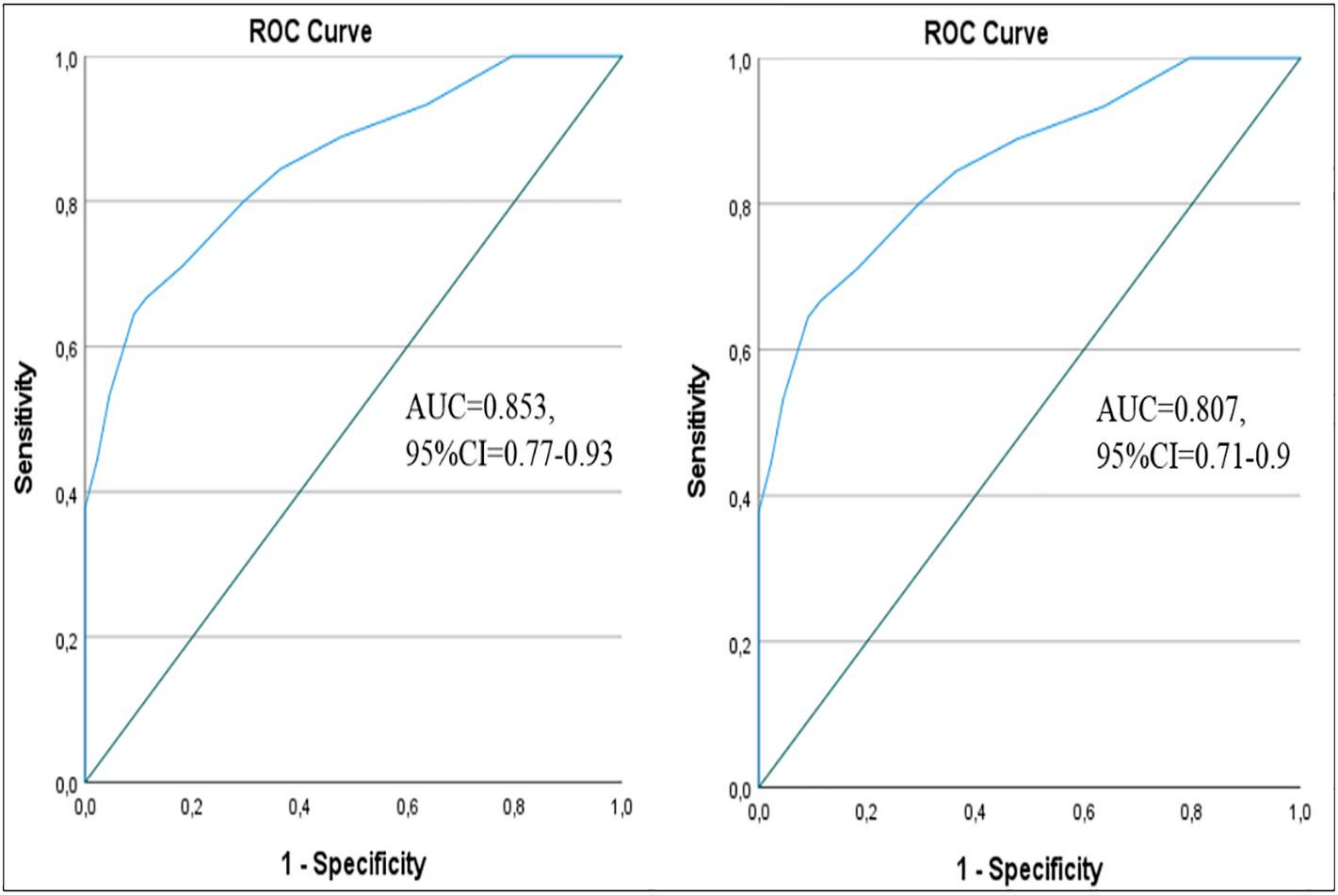
ROC curve analysis for the screening accuracy of (a) AECT‐4wk (AUC = 0.853, 95%CI = 0.77–0.93, *p* < 0.001) and (b) AECT‐3mth (AUC = 0.807, 95%CI = 0.71–0.9, *p* < 0.001). AUC, area under the curve; ROC, receiver operating characteristic.

The ability of the AECT versions for the detection of meaningful or non‐meaningful disease control was tested. In visit 1, 44 and 45 patients were categorized as having poorly‐controlled and well‐controlled disease according to LS‐AEC‐4wk and 60 and 29 patients had poorly‐controlled and well‐controlled disease according to LS‐AEC‐3mth, respectively. In the ROC curve analysis, the AUC value for the AECT‐4wk was 0.853 and the AUC value for the AECT‐3mth was 0.807, showing excellent screening accuracy for both Turkish AECT versions (Figure 1). The sensitivity and specificity of the Turkish AECT‐4wk and‐3mth to identify patients with poorly or well‐controlled disease was best for ≥10 as the cut‐off point (sensitivity:71.1%; specificity: 81.8%; Table 3). This cut‐off was also best for the AECT‐3mth (sensitivity: 85%; specificity: 65.5%) These results confirm that AECT scores equal or greater than 10 indicate well‐controlled disease.

**TABLE 3:** Sensitivity and specificity for various cut‐off points of the Turkish AECT‐4wk and ‐3mth to discriminate poorly‐and well‐controlled disease in patients with recurrent angioedema.

AECT‐4wk	Sensitivity	Specificity	AECT‐3mth	Sensitivity	Specificity
≥4	1.000	0.136	≥4	0.167	1.000
≥5	1.000	0.205	≥5	0.317	0.966
≥6	0.933	0.364	≥6	0.417	0.897
≥7	0.889	0.523	≥7	0.550	0.862
≥8	0.844	0.636	≥8	0.650	0.793
≥9	0.800	0.705	≥9	0.800	0.655
≥10	0.711	0.818	≥10	0.850	0.655
≥11	0.667	0.886	≥11	0.900	0.483
≥12	0.644	0.909	≥12	0.933	0.448
≥13	0.533	0.955	≥13	0.950	0.241
≥14	0.444	0.977	≥14	0.983	0.241
≥15	0.378	1.000	≥15	0.983	0.103
≥16	0.244	1.000	≥16	0.983	0.034

For the Turkish AECT‐4wk, the median number of attacks in the previous month and the median scores of AAS‐28 day, VAS‐AEC‐4wk, and AE‐QoL were higher whereas the median MCS‐12 and PCS‐12 scores were lower in patients with poorly‐controlled disease than with well‐controlled disease (for number of attack *p* = 0.001, for PCS‐12 *p* = 0.003, for others *p* < 0.001). Also, the number of patients whose QoL was moderately or severely affected according to AE‐QoL was higher and the number of patients who reported sufficient treatment was lower in patients with poorly‐controlled as compared to well‐controlled disease (*p* < 0.001). For the AECT‐3mth, the median number of attacks in the last 3 months and the median scores of VAS‐AEC‐3mth and AE‐QoL were higher and the median score of PCS‐12 was lower in patients with poorly controlled RAE as compared to well‐controlled RAE (for number of attack *p* = 0.006, for VAS‐AEC‐3mth, AE‐QoL and PCS‐12 *p* < 0.001). Although the median MCS‐12 score was numerically lower in patients with poorly‐controlled disease, it was not statistically significantly different as compared to patients with well‐controlled disease (*p* = 0.053). The number of patients who reported sufficient treatment was lower, whereas the number of patients with moderately or severely affected QoL was higher in patients with poorly‐controlled disease than in those with well‐controlled disease (*p* < 0.001, *p* = 0.003) (Table 4). Taken together, these results demonstrate that Turkish AECT scores equal or greater than 10 indicate well‐controlled disease.

**TABLE 4:** Comparison of number of disease activity, impairment in QoL and treatment sufficiency between patients with poorly‐ and well‐controlled disease.

	AECT‐4wk			AECT‐3mth		
	Poorly controlled *n* = 44 49.4%	Well controlled *n* = 45 50.6%	*p*	Poorly controlled *n* = 60 67.4%	Well controlled *n* = 29 32.6%	*p*
Number of AE attack^a^, median (IQR)	4 (3–7)	1 (0–5)	0.001	12 (8–22.25)	5 (2.25–11)	0.006
AAS‐28 day, median (IQR)	31 (20–51)	8 (0–26)	<0.001	‐	‐	‐
VAS‐AEC^b^, median (IQR)	70 (50–80)	10 (0–50)	<0.001	62.5 (50–80)	45 (16.25–67.5)	<0.001
AE‐QoL, median (IQR)	50 (33–60.29)	24 (10.29–42)	<0.001	43 (29–60)	22.5 (7–46.68)	<0.001
Level of impairment in QoL, *n* (%)						
No/mild impairment of QoL, *n* (%)	11 (25%)	33 (73.3%)	<0.001	23 (38.3%)	21 (72.4%)	0.003
Moderate/severe impairment of QoL, *n* (%)	33 (75%)	12 (13.5%)		37 (61.7%)	8 (27.6%)	
PCS‐12, median (IQR)	40 (35–49.61)	49.57 (41.6–54.4)	0.003	40.25 (34.5–49.4)	52 (47.2–54.8)	<0.001
MCS‐12, median (IQR)	36.6 (32–46)	50.4 (39–56.7)	<0.001	39.8 (32.4–49.9)	46.9 (36.5–57.2)	0.053
Sufficient treatment, *n* (%)	16 (36.4%)	33 (73.3%)	<0.001	17 (28.3%)	19 (65.5%)	<0.001

Abbreviations: AAS‐28, angioedema activity score for 28 days; AE, angioedema; AE‐QoL, angioedema quality of life; IQR, inter quartile range; VAS‐AEC, visual analogue scale for angioedema control.

^a^ For AECT‐4wk and AECT‐3mth the number of attacks was counted in the last month and in the last 3 months, respectively.

^b^ For AECT‐4wk and AECT‐3mth VAS‐AEC‐4wk and VAS‐AEC‐3mth were analyzed, respectively.

### 4.2 Sensitivity to change

To evaluate whether the Turkish AECT versions can measure changes in the patients' disease status, we performed correlation analysis between intraindividual changes in AECT scores and changes assessed with other tools. Change in AECT‐4wk was strongly correlated with changes in AAS‐28 day and VAS‐AEC‐4wk (*p* < 0.001, *r* = −0.602; *p* < 0.001, *r* = −0.604) and moderately correlated with changes in AE‐QoL and PCS‐12 (*p* = 0.007, *r* = −0.3; *p* = 0.009, *r* = 0.291), but was not correlated with a change in MCS‐12 (*p* > 0.05). Also, AECT‐4wk change was associated with the change in patient‐self assessment of treatment sufficiency (*p* < 0.016). Change in AECT‐3mth was moderately correlated with change in VAS‐AEC‐3mth (*p* = 0.002, *r* = −0.336) and weakly correlated with a change in AE‐QoL (*p* = 0.011, *r* = −0.282), but was not correlated with changes in PCS‐12 and MCS‐12 (*p* > 0.05). Also, change in AECT‐3mth score was not related to change in patient‐self assessment of treatment sufficiency (*p* > 0.05).

### 4.3 Minimal clinically important difference

To determine the MCID of the Turkish AECT‐4wk and AECT‐3mth, we determined their average change in 32 patients who reported relevant improvement of disease control as assessed by the LS‐AEC, over 4 weeks and 3 months.

**FIGURE 2:**
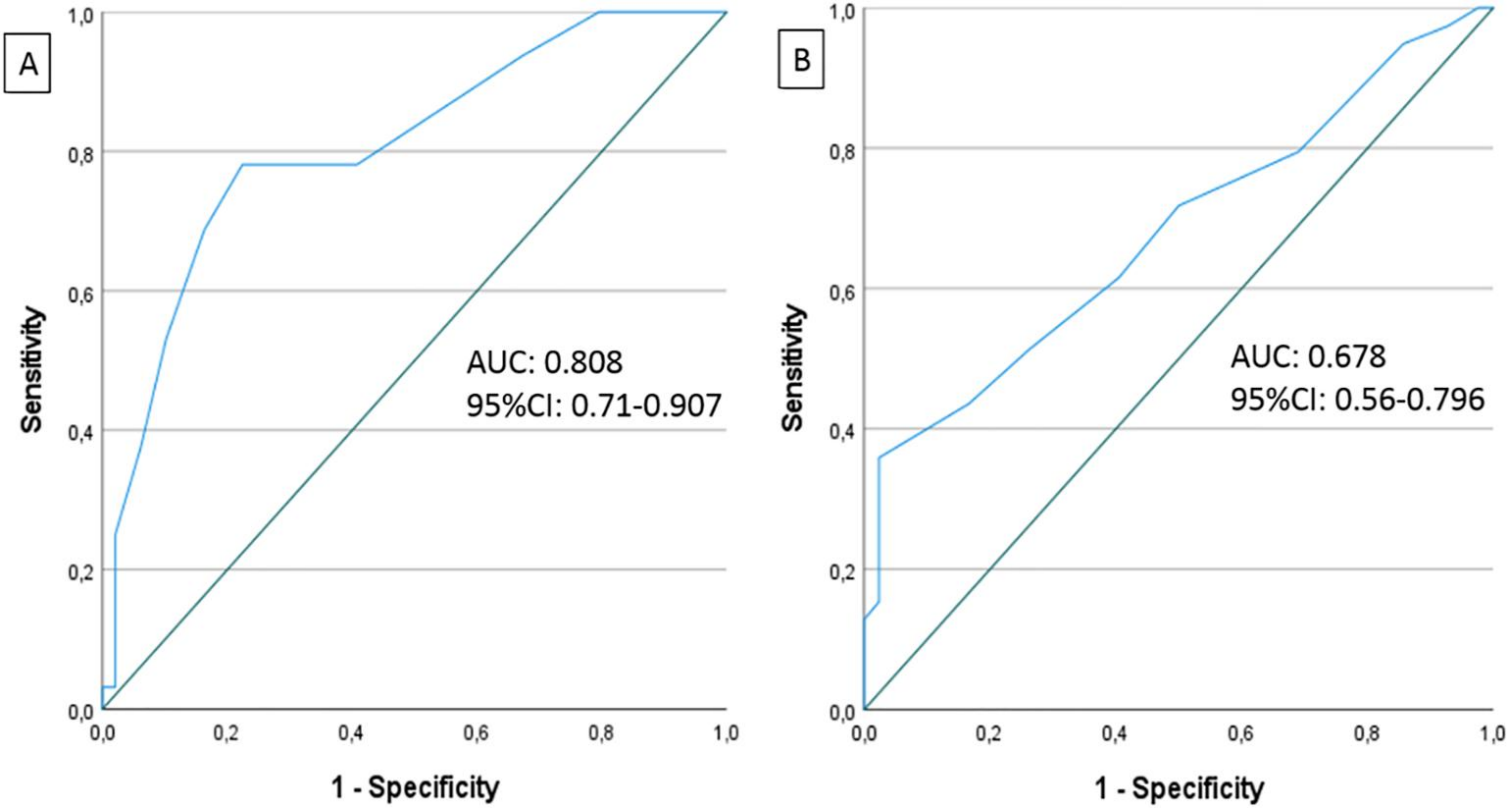
ROC curve analysis to determine the relationship between at least one step improvement in disease control level and change in (a) AECT‐4wk and (b) AECT‐3mth. AUC, area under curve; ROC, receiver operating characteristic.

The mean ± SD and median (IQR) of change in AECT‐4wk was 3.7 ± 3.0 and 4 (2–5.75), respectively (Table S2). By ROC curve analysis, the cut‐off points for meaningful AECT‐4wks improvement with the best balance of sensitivity and specificity were two and three points (Figure 2A and Table 5). When we performed the same analysis in MMAE (*n* = 22) and BMAE/HAE (*n* = 17) separately, a three‐point increase in AECT‐4wk showed 68.2% and 70% sensitivity and 83.3% and 84% specificity, respectively.

**TABLE 5:** Sensitivity and specificity for different cut‐off values of the change in the Turkish AECT‐4wk and ‐3mth versions leading to meaningful improvement, respectively.

Change in AECT‐4wk	Sensitivity	Specificity	Change in AECT‐3mth	Sensitivity	Specificity
≥1	0.781	0.592	≥1	0.718	0.500
≥2	0.781	0.776	≥2	0.615	0.595
≥3	0.688	0.837	≥3	0.513	0.738
≥4	0.531	0.898	≥4	0.436	0.833
≥5	0.375	0.939	≥5	0.359	0.976
≥6	0.250	0.980	≥6	0.256	0.976
≥7	0.188	0.980	≥7	0.154	0.976
≥8	0.063	0.980	≥8	0.128	1.000
≥9	0.031	0.980	≥9	0.077	1.000
≥10	0.031	1.000	≥10	0.026	1.000

As for the AECT‐3mth, 39 patients with meaningful improvement in LS‐AEC‐3mth, had a mean ± SD and median (IQR) change of 3.02 ± 3.48 and 3 (0–6), respectively (Table S2). Equal or greater than 2 and 3‐point increases in AECT‐3mth showed the best sensitivity (61.5% and 51.3%) and specificity (59.5% and 73.8%) (Figure 2B and Table 5). In separate analyses of patients with MMAE (*n* = 22) and BMAE/HAE (*n* = 10) 3‐point increases in AECT‐3mth detected meaningful improvement with 68.2% and 70.8% sensitivity and 83.3% and 87.5% specificity, respectively.

### 4.4 Comparison of AECT‐4wk and ‐3mth

**FIGURE 3:**
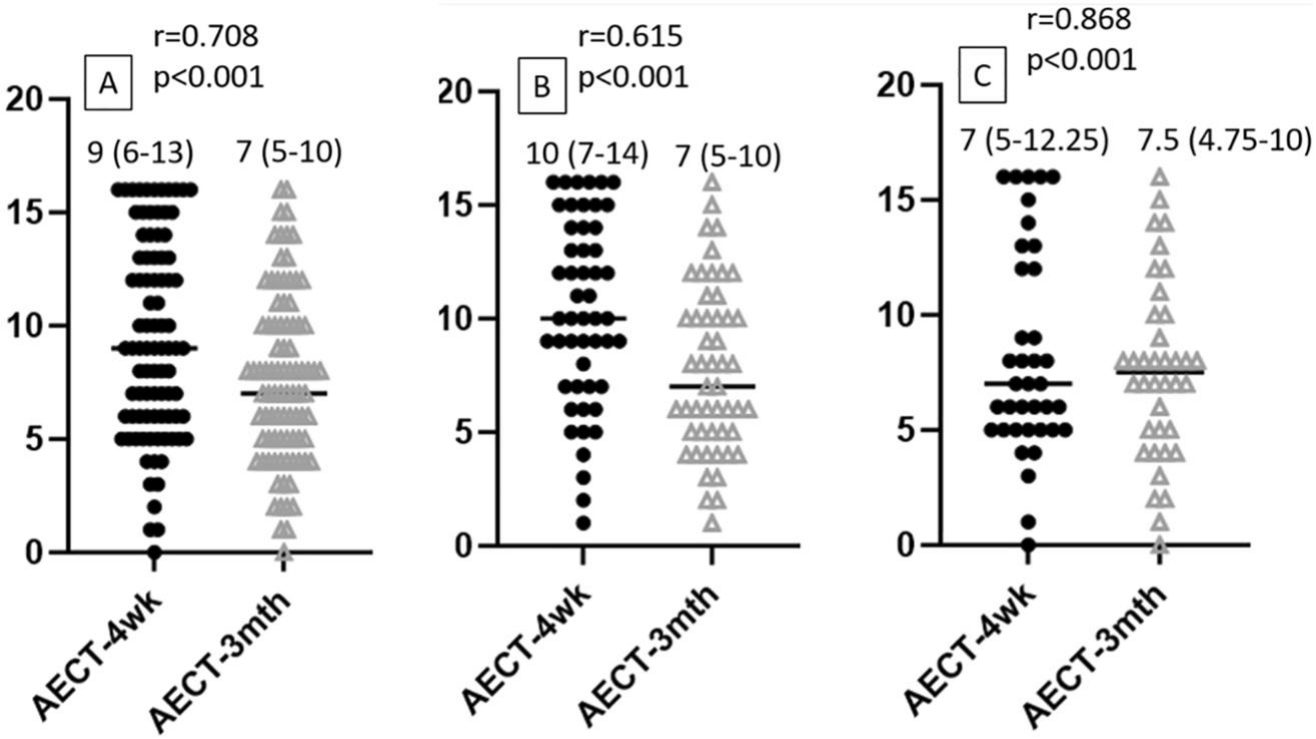
Correlation between AECT‐4wk and ‐3mth scores in (a) all patients, (b) patients with MMAE and (c) with HAE. HAE, hereditary angioedema; MMAE, mast cell‐mediated angioedema.

The AECT‐4wk and ‐3mth scores were strongly correlated with each other in all patients with RAE as well as in patients with MMAE and HAE (Figure 3).

The median AECT‐4wk was lower and VAS‐AEC‐4wk was higher in patients with HAE than in those with MMAE (*p* = 0.018, *p* = 0.001), whereas the other scores were similar (Table S1).

## 5 DISCUSSION

The current study comprising patients with two major RAE types indicates that the Turkish AECT‐4wk and‐3mth versions are valid and reliable instruments for the assessment of control of RAE. Importantly, our study is the first to evaluate the sensitivity to change and the MCID of the AECT‐3mth and it provides novel insights on the clinicometric properties of the AECT in different RAE types.

The current study indicated that both the Turkish AECT‐4wk and ‐3mth versions have a good level of convergent validity. Both versions showed strong correlation with AE‐QoL and VAS‐AEC scores and moderate correlation with SF‐12 components and number of attacks. AECT‐4wk also had a strong correlation with AAS‐28 day. Since AE‐QoL is a disease specific measure of impairment in QoL while SF‐12 is a nonspecific tool, this fact could explain the difference in the power of correlation between AECT and AE‐QoL or SF‐12 components. Similarly, in the original validation study, correlation with SF‐12 was lower than the correlation with AE‐QoL.^10^ In the Thai version, similar to our study, a good level of correlation with AE‐QoL, AAS‐28 day and numeric control scales was reported.^21^ Also, the Turkish AECT‐4wk and ‐3mth had a high known‐groups validity level, indicating that AECT can discriminate patients with various disease states in concordance with the original and Thai versions.^10^, ^21^ The current study showed that the Turkish AECT versions were reliable tools with excellent internal consistency and reproducibility in line with the other versions.^10^, ^21^

We observed a good level of screening accuracy in concordance with the original and Thai versions.^10^, ^21^ In the original study, it was found that equal or greater than 10 points showed well‐controlled disease with 75.5% and 81.8% sensitivity and 83.9% and 91.7% specificity for the AECT‐4wk and ‐3mth, respectively.^10^ In the Thai study, the cut‐off point to determine well‐controlled disease was similarly equal or more than 10 points with 97.2% and 82.8% sensitivity and 88.9% and 77.8% specificity for the AECT‐4wk and ‐3mth, respectively.^21^ We also observed that the best cut‐off point seemed to be 10 with 71.1% and 85% sensitivity and 81.8% and 65.5% specificity for the AECT‐4wk and ‐3mth, respectively. Although the cut‐off point was the same in all versions, our findings regarding sensitivity and specificity were more compatible with the original German versions than the Thai version, which could be explained by the geographical and ethnical variations. Then, we showed that the number of attacks, AAS‐28 day (for AECT‐4wk), VAS‐AEC and AE‐QoL scores were higher and PCS‐12 and MCS‐12 scores were lower in patients with poorly‐controlled (AECT ≤ 9). Also, moderately or severely impairment in QoL and insufficient treatment were prominent in patients with poorly‐controlled disease. These findings indicated that AECT is concordant with other valid and reliable PROMs, supporting our result. Similar findings were also reported in the original study, while these analyses were not performed in the Thai study.^10^, ^21^ Consequently, it is clear that an AECT score equal or less than 9 can detect poorly‐controlled disease to aid the treatment decision.

In the present study, we analyzed the sensitivity to change and MCID of AECT, as well. Very recently, Fijen et al. reported that the strength of sensitivity to change of AECT‐4wk to detect the change in disease control determined by physicians, angioedema related QoL and patient rated treatment sufficiency was high while it was lower in the detection of change in patients‐self assessment of disease control. However, they did not analyze the correlation between change in AECT‐4wk and change in disease activity and sensitivity to change in AECT‐3mth.^11^ We found that changes in AECT‐4wk were sensitive to detect the changes in disease activity, disease control assessed by patients and treatment sufficiency, while less sensitive to detect the changes in AE‐related QoL. The sensitivity to change of AECT‐3mth to detect the change in disease control and AE‐Qol was weak. Furthermore, correlations between changes in AECT versions and changes in SF‐12 components were not substantial. Fijen et al. also reported a low level of correlation between the change in AECT‐4wk and dermatology life quality index (DLQI).^11^ This can be explained by the fact that neither SF‐12 nor DLQI are not disease specific instruments.

MCID is an important property of PROMs since it is the ability to detect clinically important changes in the patient's status over a period. Fijen et al. reported that 3‐point change in AECT‐4wk can detect the minimal clinically important improvement in the disease control level of patients with RAE with a sensitivity of 86% and a specificity of 91%.^11^ We found that at least 2‐point increase in AECT over 4 weeks could detect a meaningful improvement in disease control with 78.1% sensitivity and 77.6% specificity and at least 3‐3‐point increase with lower sensitivity (68.8%) but better specificity (83.7%). Furthermore, we assessed the MMAE and HAE disease separately for the first time and found that 3‐point increase in AECT‐4wk can identify the minimal improvement in disease control in both patients with MMAE and HAE with a specificity of 83.3% and 84%, respectively. In our study, we analyzed the MCID of AECT‐3mth for the first time and showed that the 3‐point increase in AECT‐3mth can identify a meaningful improvement in disease control with 73.8% specificity. In HAE and MMAE, the specificity of 3‐point increase in AECT‐3mth was 87.5% and 55.6%, respectively. Therefore, we can say that the 3‐point increase in AECT‐4wk is a more favorable tool to determine the improvement in the disease control in all patients independent of the underlying diseases. On the other hand, 3‐point increase in AECT‐3mth can be an indicator of meaningful improvement in control of HAE more specifically than in control of MMAE.

Regarding all the results, we can present that AECT‐4wk can be a more accurate tool than the AECT‐3mth to determine the control level of the disease even though we observed strong correlations between AECT‐4wk and ‐3mth in all patients and also in patients with either HAE or MMAE. However, the other authors in Thai versions reported that AECT‐4wk may be preferable to the AECT‐3mth.^21^

As a limitation, we could not determine the impact of AECT on the deterioration of the disease control since we did not have enough patients with deterioration in disease control. Furthermore, we did not include other types of RAE such as idiopathic or acquired AE because they are rarely seen compared to HAE and MMAE and we anticipated that we could not obtain enough data during the study period to compare other groups.

To conclude, we found that the Turkish AECT versions were valid and reliable tools for the monitoring of disease control in patients with RAE. Equal or more than 10 points in AECT can indicate well‐controlled disease. Three‐point chance in AECT‐4wk and ‐3mth scores can represent a minimal improvement in disease control level in patients with RAE. AECT‐4wk seems to be more appropriate than the AECT‐3mth for the evaluation of control and to detect the minimal clinically important change in RAE. We believe that the findings of our study will add to the utility of AECT in clinical practice.

## AUTHOR CONTRIBUTIONS

Semra Demir, Deniz Eyice Karabacak, Derya Ünal, İlkim Deniz Toprak, Pelin Korkmaz, Ayşe Feyza Arslan, Işıl Göğem İmren, Bahar Dikicier, Nevzat Kahveci, Nida Öztop, Rabia Öztaş Kara and Aslı Gelincik made substantial contributions to conception and design, acquisition of data, and analysis and interpretation of data; and were involved in drafting the manuscript or revising it critically for important intellectual content; and gave final approval of the version to be published. Emek Kocatürk, Marcus Maurer and Karsten Weller made substantial contributions to conception and design and interpretation of data, were involved in revising the manuscript critically for important intellectual content, and gave final approval of the version to be published. Each author participated sufficiently in the work to take public responsibility for appropriate portions of the content; and agreed to be accountable for all aspects of the work in ensuring that questions related to the accuracy or integrity of any part of the work are appropriately investigated and resolved.

## CONFLICT OF INTEREST STATEMENT

The authors declare that they have no relevant or material financial interests that relate to the data and research described in this publication. Emek Kocatürk has received honoraria from Novartis, Menarini, Pfizer, Sanofi, La Roche Posey and Abdi İbrahim. Bahar Sevimli Dikicier has received honoraria from Novartis. Marcus Maurer or recently was a speaker and/or advisor for and/or has received research funding from Allakos, Allvotech, Amgen, Aquestive, Aralez, AstraZeneca, Astria, Bayer, BioCryst, Celldex, Celltrion, CSL Behring, Evommune, GSK, Ipsen, Kalvista, Leo Pharma, Lilly, Menarini, Mitsubishi Tanabe Pharma, Moxie, Noucor, Novartis, Orion, Biotechnology, Pharvaris, Resonance Medicine, Sanofi/Regeneron, Septerna, Takeda, Teva, Third HarmonicBio, Trial Form support International AB, ValenzaBio, Yuhan Corporation and Zurabio. Karsten Weller is or was recently an advisor for Novartis and has received honoraria from Moxie, Takeda, Novartis, CSL Behring and Biocryst.

## Supporting information

Supporting Information S1

## Data Availability

Research data are not shared.
