# Supplementary material for: Monitoring recurrent angioedema: Findings from the Turkish angioedema control test validation study
Source: Clin Transl Allergy. 2024 Feb 28;14(3):e12342. doi: 10.1002/clt2.12342 (PMC10900914; doi:10.1002/clt2.12342)
Supplement: Supplementary file 1 — Supporting Information S1 [file CLT2-14-e12342-s001.docx]

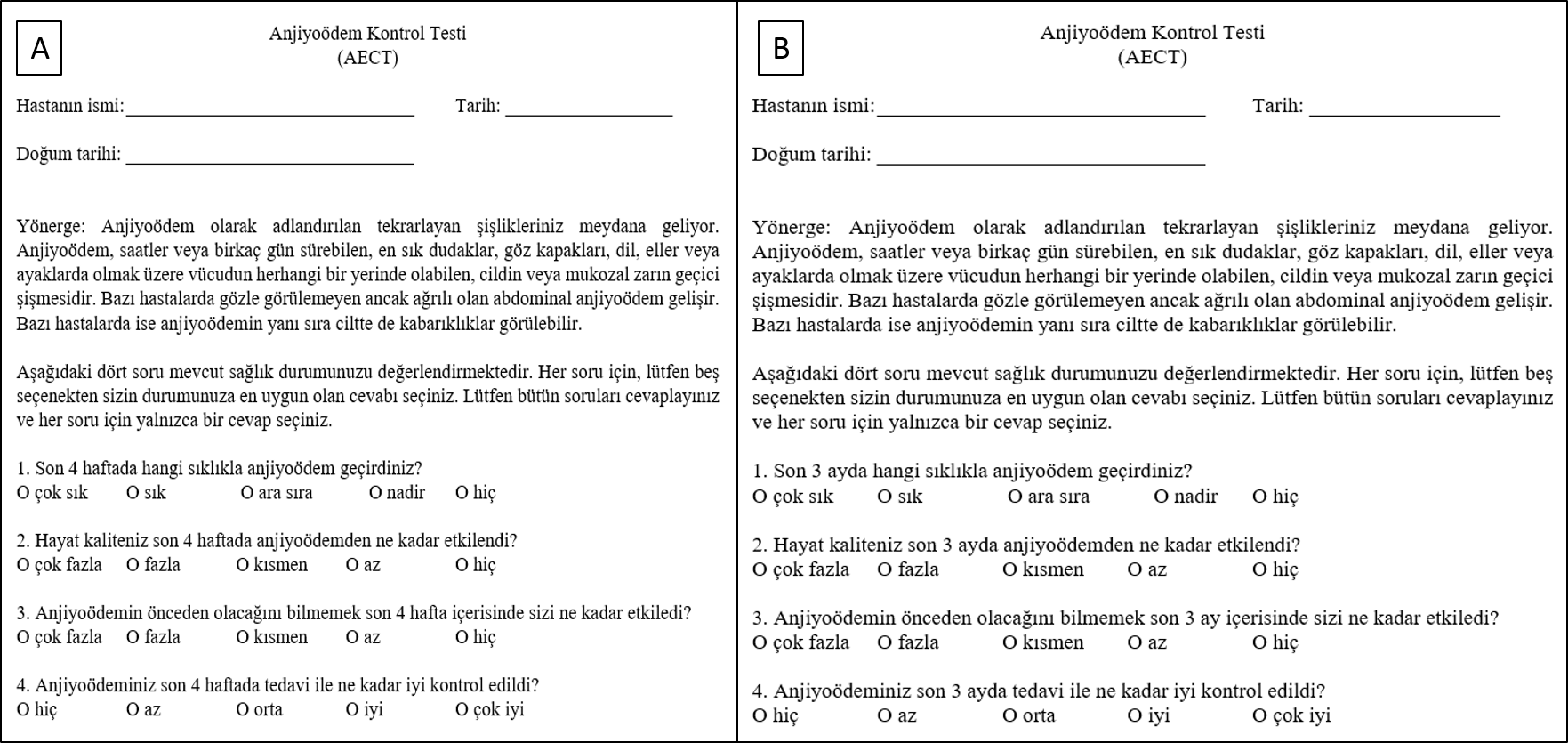


Supplement Figure 1. A) Turkish version of AECT-4wk, B) Turkish version of AECT-3mth

Supplement Table 1: Demographic and clinical features of the patients with MMAE and HAE

| **Features** | **MMAE**  **n=55 (57.3%)** | **HAE**  **n=38 (42.7%)** | **p** |
| --- | --- | --- | --- |
| Age, year [median (IQR)] | 37 (26-53) | 31 (22-40.25) | >0.05 |
| Gender (n, %) |  |  |  |
| Female | 41 (80.4%) | 23 (60.5%) | 0.039 |
| Male | 10 (19.6%) | 15 (39.5%) |  |
| Education level |  |  |  |
| Primary-middle school | 12 (24.5%) | 6 (15.8%) |  |
| High school | 16 (32.7%) | 9 (23.7%) | >0.05 |
| University and higher | 21 (42.9%) | 23 (60.5%) |  |
| Being smoker (n, %) | 10 (20.4%) | 11 (28.9%) | >0.05 |
| Disease o duration, months [median (IQR)] |  |  |  |
| Since onset of symptoms | 1 (1-3) | 60 (1-189) | 0.004 |
| Since diagnosis | 4 (1-12) | 26 (1-144) | 0.003 |
| Location of RAE, ever (n,%) |  |  |  |
| Larynx/pharynx | 17 (34.7%) | 22 (57.9%) | 0.031 |
| Tongue | 14 (28.6%) | 14 (36.8%) | >0.05 |
| Uvula | 8 (16.3%) | 14 (36.8%) | 0.029 |
| Periorbital | 36 (73.5%) | 11 (28.9%) | <0.001 |
| Lips/cheeks/chin | 45 (91.8%) | 29 (76.3%) | >0.05 |
| Extremities | 32 (65.3%) | 38 (100%) | <0.001 |
| Genital | 13 (26.5%) | 25 (65.8%) | <0.001 |
| Presence of familial RAE history (n, %) | 7 (14%) | 30 (78.9%) | <0.001 |
| Presence of comorbid diseases (n, %) | 31 (60.8%) | 9 (23.7%) | 0.001 |
| Number of AE attacks in the previous month | 3 (1-7) | 3 (1-5) | >0.05 |
| Number of AE attacks in the previous 3 months | 11 (5-25) | 8.5 (4-14.25) | >0.05 |
| **At visit 1** |  |  |  |
| AECT-4 week, [median (IQR)] | 10 (8-14) | 7 (5-12.75) | 0.018 |
| AECT-3 month, [median (IQR)] | 7 (5-10) | 7.5 (5-10) | >0.05 |
| AE-QoL, [median (IQR)] | 32.35 (20.6-54) | 41.2 (16.5-59.7) | >0.05 |
| VAS-AEC-4 week, [median (IQR)] | 35 (5-60) | 60 (30-77.5) | 0.001 |
| VAS-AEC-3 month, [median (IQR)] | 60 (40-80) | 55 (42.5-70) | >0.05 |
| LS-AEC-4 week |  |  |  |
| Poorly-controlled disease, n (%) | 22 (43.1%) | 22 (57.9%) | >0.05 |
| Well-controlled disease, n (%) | 29 (56.9%) | 16 (42.1%) |  |
| LS-AEC-3 month |  |  |  |
| Poorly-controlled disease, n (%) | 35 (68.6%) | 25 (65.8%) | >0.05 |
| Well-controlled disease, n (%) | 16 (31.4%) | 13 (34.2%) |  |
| AAS-28 day, [median (IQR)] | 20 (5-49) | 15 (5.75-37.75) | >0.05 |
| PCS-12, [median (IQR)] | 48.6 (35.9-53.7) | 46.6 (39.84- 51) | >0.05 |
| MCS-12, [median (IQR)] | 43 (33.6-51) | 44.23 (33.47-55.5) | >0.05 |
| Sufficient treatment (PSA) (n, %) | 16 (31.4%) | 20 (52.6%) | 0.043 |
| Physicians’ decision on treatment |  |  | 0.006 |
| No change (n, %) | 27 (52.9%) | 32 (84.2%) |  |
| Step-up (n, %) | 19 (37.3%) | 5 (13.2%) |  |
| Step-down (n, %) | 5 (9.8%) | 1 (2.6%) |  |

Supplement Table 2: Magnitude of changes in AECT-4wk and -3mth scores in patients with unchanged, improved or worsened at least one step according to LS-AEC

| Disease control according to LS | Change in AECT-4wk | | | Change in AECT-3mth | | |
| --- | --- | --- | --- | --- | --- | --- |
|  | Number of patients | Mean±SD | Median (IQR) | Number of patients | Mean±SD | Median (IQR) |
| Improved at least one step | 32 | 3.71±2.96 | 4 (2-5.75) | 39 | 3.02±3.48 | 3 (0-6) |
| Unchanged | 37 | 0.18±2.2 | 0 (-1-1) | 27 | 0.77±2.2 | 0 (-1-2) |
| Worsened at least one step | 12 | -0.33±4.73 | 0.5 (-4.5-1.75) | 15 | 1±2.72 | 1 (-1-4) |
